# Bibliometric analysis of MXRA7 gene research trajectory: trends and insights (2015–2024)

**DOI:** 10.1007/s12672-025-02824-5

**Published:** 2025-06-03

**Authors:** Huihui Zhang, Ying Shen, Peijian Bai, Xiaorui Wu, Ping Li, Ting Wang

**Affiliations:** 1 Oncology Department of Integrated Traditional Chinese and Western Medicine, The First Affiliated Hospital of Anhui Medical University, Hefei, 230001 China; 2 National Clinical Research Center for Hematologic Disease, Institute of Blood and Marrow Transplantation of Soochow University, The First Affiliated Hospital of Soochow University, Suzhou, 215006 China

**Keywords:** Matrix remodeling-associated 7, Bibliometrics, Extracellular matrix remodeling

## Abstract

**Background:**

Matrix remodeling-associated 7 (MXRA7) plays a key role in physiological and pathological processes involving the extracellular matrix (ECM) and tissue remodeling. Recent studies have highlighted its functions in tissue injury, immune response, and cellular differentiation, yet no bibliometric studies have systematically mapped MXRA7 research. This study evaluates global MXRA7 research from 2015 to 2024 to identify current trends and future directions.

**Methods:**

A comprehensive bibliometric analysis was conducted using the Web of Science Core Collection. We examined publication trends, geographical contributions, influential authors, and high-impact journals, identifying research hotspots and emerging trends with advanced bibliometric tools.

**Results:**

Analysis of 553 English-language publications showed that MXRA7 research has progressed significantly after 2017, showing a general upward trend accompanied by short-term fluctuations. The United States leads, followed by China and the United Kingdom. Key studies appear in high-impact journals like PLOS ONE, and influential authors such as Wang Yiqiang have propelled the field. Keywords including “inflammation”, “extracellular matrix”, “matrix metalloproteinases” and “angiogenesis” underscore MXRA7’s roles in immune responses, tissue repair, and fibrosis.

**Conclusion:**

This analysis shows significant growth in MXRA7 research, especially in inflammation, ECM remodeling, and tissue regeneration. Future work should explore MXRA7’s molecular mechanisms in immune diseases, fibrosis, and cancer, advancing its potential as a therapeutic target.

## Introduction

MXRA7, a member of the matrix remodeling-associated gene family, plays a crucial role in various physiological and pathological processes related to extracellular matrix (ECM) and tissue remodeling [1, 2]. This gene family was first identified in a bioinformatics study by Walker and Volkmuth in 2002, which identified eight transcripts that tend to be co-expressed with genes known to mediate cell adhesion or ECM remodeling, such as bone morphogenetic proteins, matrix metalloproteinases (MMPs), and collagen. These transcripts were subsequently named matrix remodeling-associated genes (MXRA1-8). Research has shown that this gene family impacts various key physiological and pathological processes involving matrix remodeling [3–11], with MXRA7 being revealed through bioinformatics analysis to co-express with genes related to matrix remodeling, such as MMPs and collagen [4]. Furthermore, other members of the MXRA family are involved in significant physiological and pathological processes. For example, MXRA2 is associated with matrix degradation [8], MXRA3 is involved in actin polymerization and cell motility [6], MXRA5 plays a crucial role in inflammation and fibrosis [9], and MXRA6 is related to myofibroblast differentiation and ECM formation [5]. Stromal remodeling and genes associated with stromal remodeling play important roles in cancer progression. Stromal remodeling can affect the tumor microenvironment, thereby promoting tumor cell proliferation and invasion [12–14]. It can also break through barriers and promote tumor invasion and metastasis [15, 16]. Importantly, ECM remodeling is also involved in angiogenesis, which is critical for tumor growth and metastasis [17]. Although the MXRA gene family was identified early on, the biological functions of MXRA7 have not been fully explored until recent years, particularly regarding its diverse roles in tissue injury, immune responses, and cell differentiation.

Recent studies have indicated that MXRA7 is involved in several key physiological processes, including inflammation, wound healing, angiogenesis, and osteogenesis [2, 3, 18–20]. For instance, in a model of neovascularization, changes in MXRA7 expression were found to correlate temporally with the activity of MMPs and other matrix-associated proteins [2, 4]. Additionally, the role of MXRA7 in the differentiation of bone marrow mesenchymal stem cells (BMSCs) into osteoblasts has been validated, further highlighting its importance in skeletal development and repair [3]. In addition, it was found that MXRA7 may be involved in the treatment of some tumors [21], but a comprehensive analysis of the role of MXRA7 in cancer and its value in immunotherapy and drug resistance regulation has not been fully explored [22]. Beyond its roles in the immune system and tissue regeneration, MXRA7 has also been linked to diseases such as psoriasis, liver injury, and cancer. In a carbon tetrachloride-induced acute liver injury mouse model, Lin demonstrated that MXRA7 modulates liver tissue damage by mediating inflammatory responses and matrix remodeling [1]. Ning and colleagues discovered that MXRA7 may negatively regulate the development of psoriasis by altering the expression or redistribution of keratinocytes [18].

Despite these advances, the research on MXRA7 still does not match the attention received by other matrix remodeling genes. In recent years, with the development of high-throughput technologies and the widespread application of public databases, researchers have been able to explore the gene’s potential more deeply, resulting in a significant shift in the trajectory of MXRA7 research around 2017. Increasingly, literature suggests that MXRA7 possesses diverse biological functions and could become an important target for the treatment of diseases characterized by matrix remodeling, such as fibrosis, cardiovascular diseases, and certain cancers.

Bibliometrics employs statistical methods and visualizations to analyze research trends and extract key insights from large datasets [23–25]. CiteSpace and VOS- Viewer are currently the most commonly used bibliometric software programs [26–28]. These tools provide insight into collaboration networks among authors, institutions, and countries, as well as co-occurrence networks of keywords and journals. Therefore, bibliometric analysis can provide valuable insights into the development of a field [29, 30].

This study aims to conduct a bibliometric analysis of the literature on MXRA7 from 2015 to 2024, focusing on research trends, global publication distribution, and the exploration of cutting-edge topics. By analyzing the scientific trajectory of MXRA7, this study will provide deep insights into the current state of research and offer guidance for future investigations into the gene’s molecular mechanisms and therapeutic potential, ultimately advancing its application in disease treatment.

## Materials and methods

### Data sources and search strategy

**Fig. 1:**
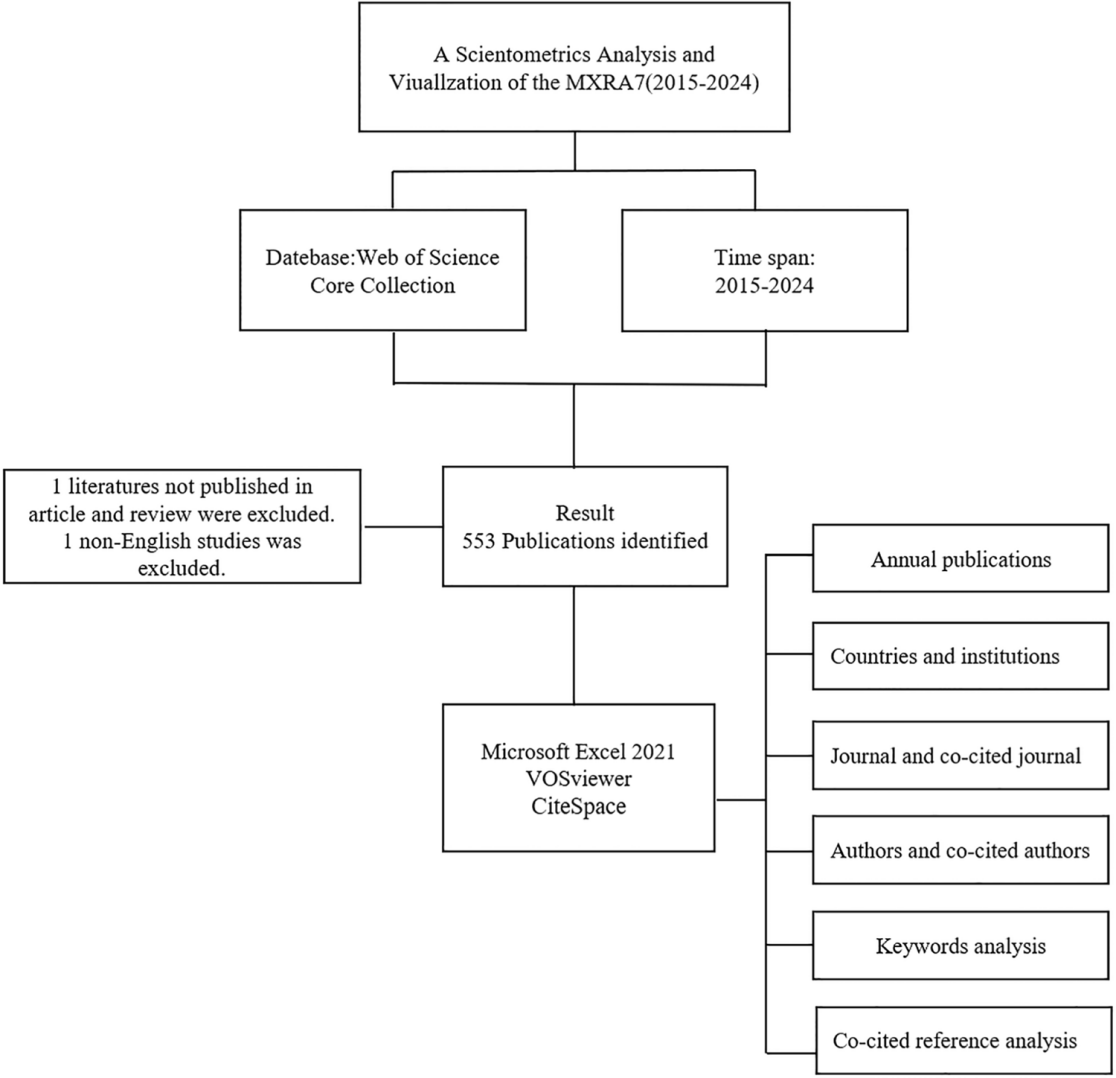
Search strategy and data processing flow for MXRA7 bibliometric analysis

The Web of Science database served as the primary source for literature collection in this study. As a widely recognized and extensively used academic database, the Web of Science Core Collection was systematically screened to identify literature meeting the specified inclusion criteria. The detailed data search strategy and inclusion process are outlined in Fig. 1**.** All searches were conducted on January 6, 2025, and independently validated by two authors to minimize potential bias from daily database updates. The search terms employed for screening included: TS = (“Matrix Remodeling Associated 7”) and TS = (“MXRA7”). The inclusion criteria were as follows: Type of Literature: Articles or Reviews, Language: English, Time Span: 2015–2024, A total of 553 articles were ultimately identified, and key information was extracted for subsequent bibliometric analysis.

### Data analysis and visualization

Before initiating analysis and visualization, bibliographic data underwent preprocessing to ensure accuracy and consistency. Duplicates were removed using CiteSpace (version 6.1.R6) through automated matching of title, author, publication year, and DOI, followed by independent manual verification by two researchers. Additional preprocessing included spelling correction and standardization of year formats. Bibliometric analyses were subsequently conducted using a range of specialized software tools. CiteSpace: Used to generate biplots of journals, keyword bursts, reference timelines, and citation bursts, providing insights into research trends and influential studies. bibliometric.com: Produced geographic maps highlighting the countries where the literature originated. VOSviewer (version 1.6.18): Created visualizations, including institutional co-occurrence graphs and author collaboration networks. In these graphs, node size and color represent citation counts and collaboration strength, respectively. R and Bibliometrix: Used to compute metrics such as citation frequency and H-index, which assess the impact and scholarly value of the literature. Bibliometrix also provided visualizations to illustrate publication trends and author collaborations. Microsoft Excel 2021: Used for analyzing annual publication trends. Web of Science (retrieved on January 10, 2025): Provided Impact Factor (IF) values and Journal Citation Reports (JCR) category classifications of journals. These tools collectively enabled a comprehensive and detailed bibliometric analysis, facilitating an in-depth understanding of the MXRA7 research landscape.

## Results and discussion

### Global trends in publications

An analysis of the 553 screened publications reveals fluctuating trends between 2015 and 2017. This variability is likely due to MXRA7’s relative obscurity during this period, as research in the field was still in its nascent and exploratory stage. However, significant breakthroughs emerged starting in 2017, particularly from Prof. Wang’s team, whose work on the MXRA7 gene in corneal injury models, liver injury models, and bone development contributed to advancing the field. High-throughput technologies and public databases accelerated research output, leading to increased publications. A brief decline in the number of publications in 2019–2021 may be that research on MXRA7 may have focused more on specific applications or more technical approaches, thus reducing the number of broad studies published in general journals. Another factor may be that researchers have begun to focus on novel applications or more targeted studies that require more rigorous experimental designs, resulting in some studies taking longer to refine, which in turn affects publication frequency.

**Fig. 2:**
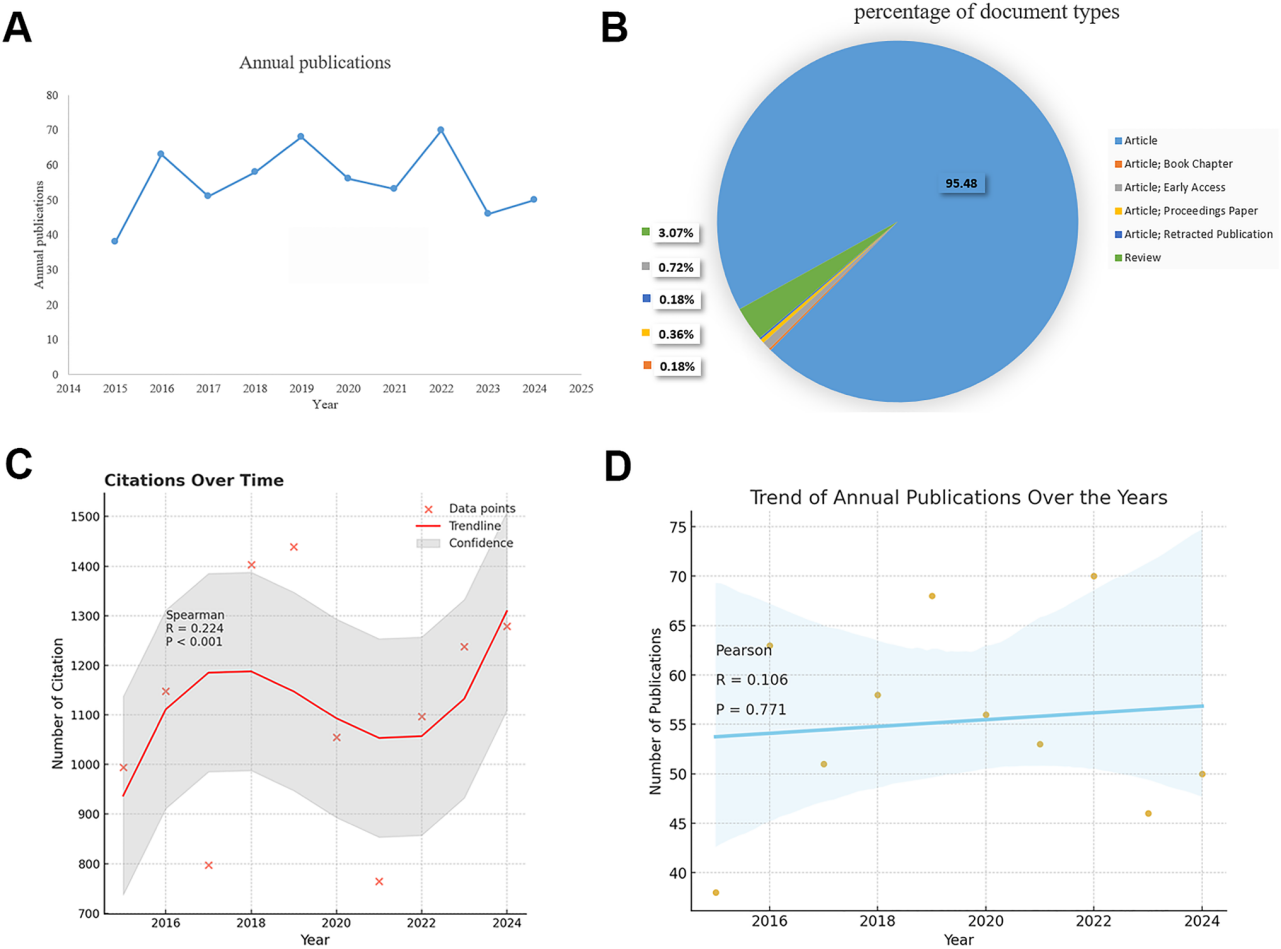
Trends in publications and citations of MXRA7-related literature between 2015 and 2024. **A** Changes in the number of publications per year. **B** Pie chart of publication types. **C** Correlation of number of citations with year. **D** Correlation of number of publications with year

The year 2021 marked the fastest growth in publication output, and 2022 recorded the highest number of publications (Fig. 2A). During this period, MXRA7’ s functions in various biological processes, such as oncology, cardiovascular disease, immune diseases, and its roles as a multifunctional matrix remodeling-associated protein, became more evident. These discoveries likely spurred significant interest, leading to increased collaborations and high-quality publications.

A short-term decline in MXRA7-related research was observed post-2022, potentially reflecting a shift toward more specialized research areas, such as specific diseases (e.g., cancer, immune diseases, cardiovascular conditions) or novel technological applications. This redistribution of focus may not indicate a decline in interest but rather a transition from broad exploratory studies to more in-depth, targeted investigations.

Of the 553 analyzed publications, 95.48% were research articles, indicating that the majority of the literature comprises original research studies (Fig. 2B). Citation analysis showed a gradual increase beginning in 2016, with the highest observed citation count recorded in 2024 based on data available as of December 31, 2024 (Fig. 2C). Although the trend line indicates a slight increase in publication numbers over time, the weak correlation suggests that publication year has a relatively small effect on overall output (Fig. 2D).

### Analysis of the distribution of issuing countries and institutions

**Fig. 3:**
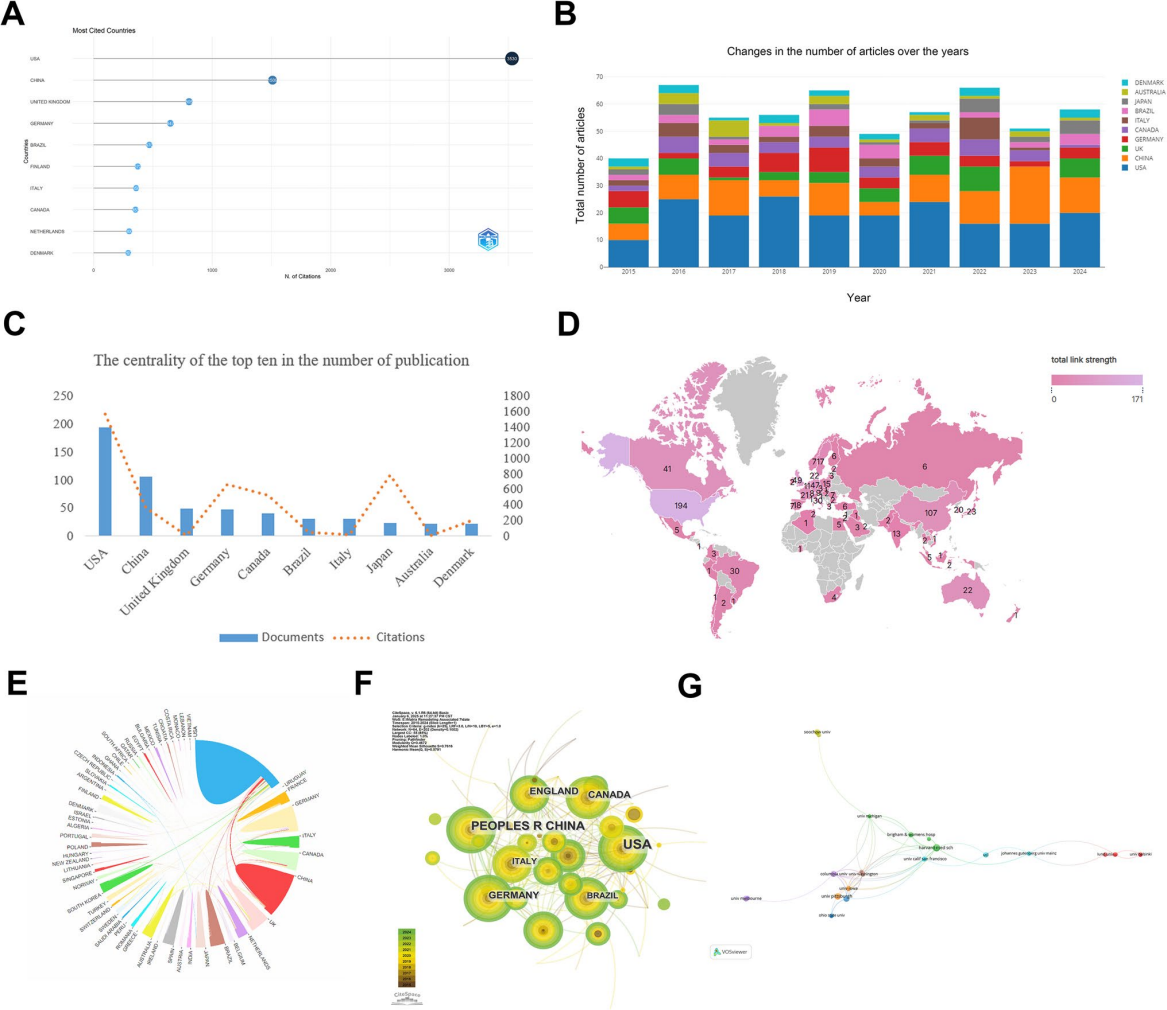
Country and institutional distribution of MXRA7-related literature. **A** Most cited countries. **B** Distribution of annual total number of literature publications and contributions by country. **C** Centrality of the number of publications and citations in the top ten countries. **D** Map of global research layout. **E** Global collaboration network. **F** Collaboration network visualization. **G** Institutions with more than 5 publications

An analysis of the published literature by country and institution highlights the United States (USA) as the most cited country in MXRA7 research, with 3,530 citations, underscoring its leadership and academic dominance in the field. China (PRC) and the United Kingdom (UK) also occupy significant positions, ranking second and third with 1,509 and 805 citations, respectively (Fig. 3A). The bar chart in Fig. 3B shows the annual distribution of publications and the contribution of countries from 2015 to 2024. In terms of the number of world publications, the total number of publications in the world gradually increases starting in 2015, with the highest number of publications in that year compared to other years in 2016. Subsequently, the number of publications in the world has remained at around 50 per year. In terms of the number of publications by country, the United States consistently leads in the number of publications in all years, accounting for about 40–50% of the total each year, demonstrating its significant academic influence. China showed a marked increase in publications, particularly after 2020, rapidly emerging as the second-largest contributor. This growth likely reflects increased research investment and international collaboration in China. The UK maintained stable contributions throughout the study period, with a slight rise in publications during 2021 and 2022, reflecting its continued relevance in global research. Germany also displayed steady output, albeit with slower growth. Collectively, the USA, China, the UK, and Germany accounted for over 50% of all publications between 2015 and 2024.

In terms of citations, the USA not only leads in publication volume but also demonstrates the highest citation count (Fig. 3C), highlighting its centrality in MXRA7 research. While China ranks second in publication volume, a significant gap in citations compared to the USA suggests differences in research quality, citation practices, or international collaboration, in addition, systematic biases-such as the dominance of English-language journals in global citation networks, the selective indexing of high-impact journals in databases such as WoS, and differences in journal impact factors-may also contribute to the gap. The United Kingdom (UK) is ranked third in terms of the number of papers published, suggesting that its contribution has been more consistent throughout the research period. However, its low number of citations may reflect a focus on particular areas of research or differences in journal preferences. Traditional research powerhouses, including the Germany, Canada, and Japan, maintained stable contributions in both publication and citation metrics. Notably, Japan, a key research hub in Asia, demonstrated consistent output (23 publications), reflecting its established strength in this field. Brazil, with 30 publications, emerged as the leading contributor in Latin America, showcasing its regional influence, while Australia, with 22 publications, highlighted its academic strength and active international cooperation (Fig. 3D).

Global collaboration networks, depicted in Fig. 3E and F, reveal the USA as the largest node, reflecting its pivotal role in fostering international partnerships. China and the UK also exhibit prominent nodes, emphasizing their significant contributions to the field. Among institutions, Harvard Medical School, the University of Michigan, and Soochow University each produced more than five publications, underscoring their active participation in MXRA7 research. US institutions, such as Harvard Medical School and the University of California, San Francisco, serve as key hubs within the collaboration network. Meanwhile, institutions from other countries, such as Soochow University (China) and Lund University (Sweden), play critical roles in advancing international collaboration (Fig. 3G).

### Journal and co‑cited journal analysis

**Fig. 4:**
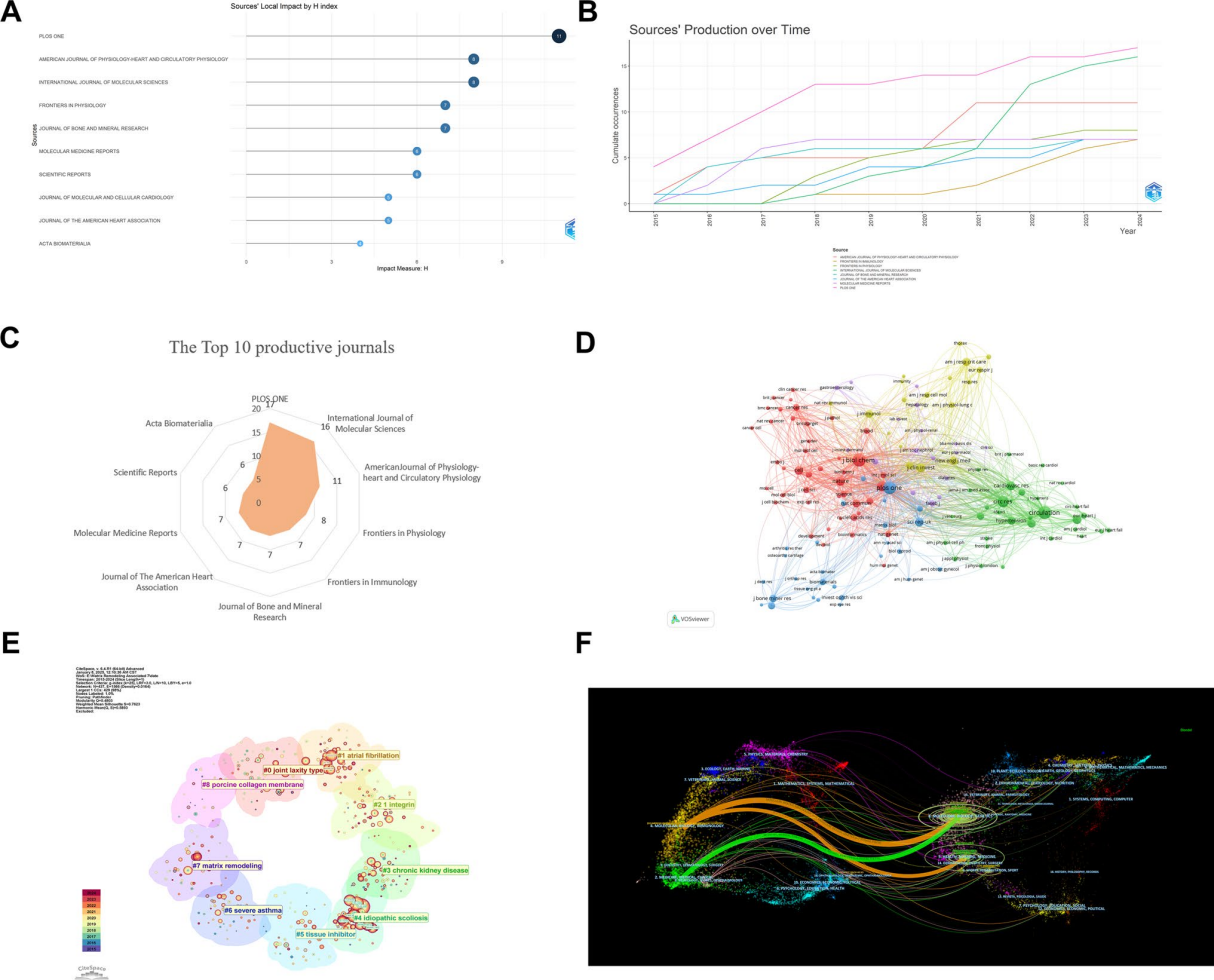
Journal distribution and co-citation relationship of MXRA7-related literature between 2015 and 2024. **A** Local impact of journals (sorted by H-index). **B** Output of journals over time. **C** Radar chart of the top 10 most productive journals. **D** Correlation network between journals with more than 40 co-citations. **E** Co-citation cluster analysis of journals. **F** Dual journal overlay graph

A total of 553 relevant papers were published across 339 journals, with PLOS ONE emerging as the most influential journal in the field. It exhibited the fastest growth in publications, the highest number of publications (17), and an H-index of 11, significantly outpacing other journals. This suggests that PLOS ONE has consistently published a substantial number of critical studies in MXRA7 research. Other highly productive journals include the International Journal of Molecular Sciences, Journal of the American Heart Association, and Scientific Reports. Different journals have different H-index performance due to their disciplinary positioning, research focus and specialisation. For example, journals with H-index 8 are American Journal of Physiology Heart And Circulatory Physiology, International Journal of Molecular Sciences, which have strong expertise and significant influence in the fields of cardiac circulation physiology and molecular sciences, and Journal of Bone And Mineral Research, with H-index 7, which is in the field of bone and mineral sciences, which is different from the multidisciplinary comprehensiveness of PLOS ONE (Fig. 4A–C).

Figure 4D depicts the network of associations among journals that have been co-cited more than 40 times. At the center of this network are the core journals Circulation, J Biol Chem (The Journal of Biological Chemistry) and PLOS ONE, with a co-citation frequency of 556, 555 and 547, respectively. reflecting their status as the most co-cited journals in the field. Other notable high-frequency co-citation journals include Nature and Science, with co-citation frequencies of 260 and 140, respectively, highlighting their authority and influence in MXRA7 research. Additional significant journals such as Cell, Nat Commun (Nature Communications), and Circ Res (Circulation Research) also exhibit high citation frequencies, further emphasizing their relevance. Journal impact needs to be assessed by combining multi-dimensional metrics: the high publication volume of PLOS ONE reflects its role as an open science platform for MXRA7 research, while the co-cited core status of J Biol Chem highlights its authority in molecular mechanism research. Both represent different levels of knowledge dissemination and integration in the field.

The co-citation network is characterized by thematic clusters, with nodes of different colors representing distinct research themes or subfields: Red Cluster (Biology and Cell Research): Includes J Biol Chem, Nature, Science, and Cell, reflecting a concentration of journals in basic biology and cell-related research. Green Cluster (Cardiovascular Research): Comprises Circulation, Circ Res, and Eur Heart J (European Heart Journal), focusing on cardiovascular medicine. Blue Cluster (Bone and Biomaterials Research): Contains J Bone Miner Res (Journal of Bone and Mineral Research) and Biomaterials, emphasizing bone and biomaterials research. Yellow Cluster (Respiratory and Immune Research): Includes Am J Respir Crit Care (American Journal of Respiratory and Critical Care Medicine) and J Immunol (Journal of Immunology), highlighting research on immune and respiratory-related topics. The co-citation relationships further underscore key journal pairings: J Biol Chem exhibits strong co-citation ties with Nature and Science, indicating these journals’ significant roles in basic science research. Circulation and Circ Res demonstrate strong co-citation connections, underscoring their prominence as core journals in cardiovascular research. Figure 4E highlights recent trends in journal topics (2020–2024), with certain areas receiving increased attention, as indicated by red-circled nodes. Notable topics include: Atrial Fibrillation (#1): Likely associated with studies on the mechanisms underlying cardiovascular diseases and related therapeutic approaches. Matrix Remodeling (#7): Involves research on tumor microenvironments, tissue repair, fibrosis, and other related fields.

In order to reveal the association between the citing and cited literature, we use CiteSpace’s dual-map overlay feature for our analysis. The method visualises interdisciplinary citation relationships by clustering citing journals (left) with cited journals (right) by discipline category. Figure 4F show that studies in molecular biology, genetics, health, nursing, and medicine are predominantly cited by journals specializing in molecular biology, immunology, and medicine. This highlights the interdisciplinary nature of MXRA7 research, bridging foundational biological sciences with clinical applications.

### Literature and co‑cited literature analysis

**Fig. 5:**
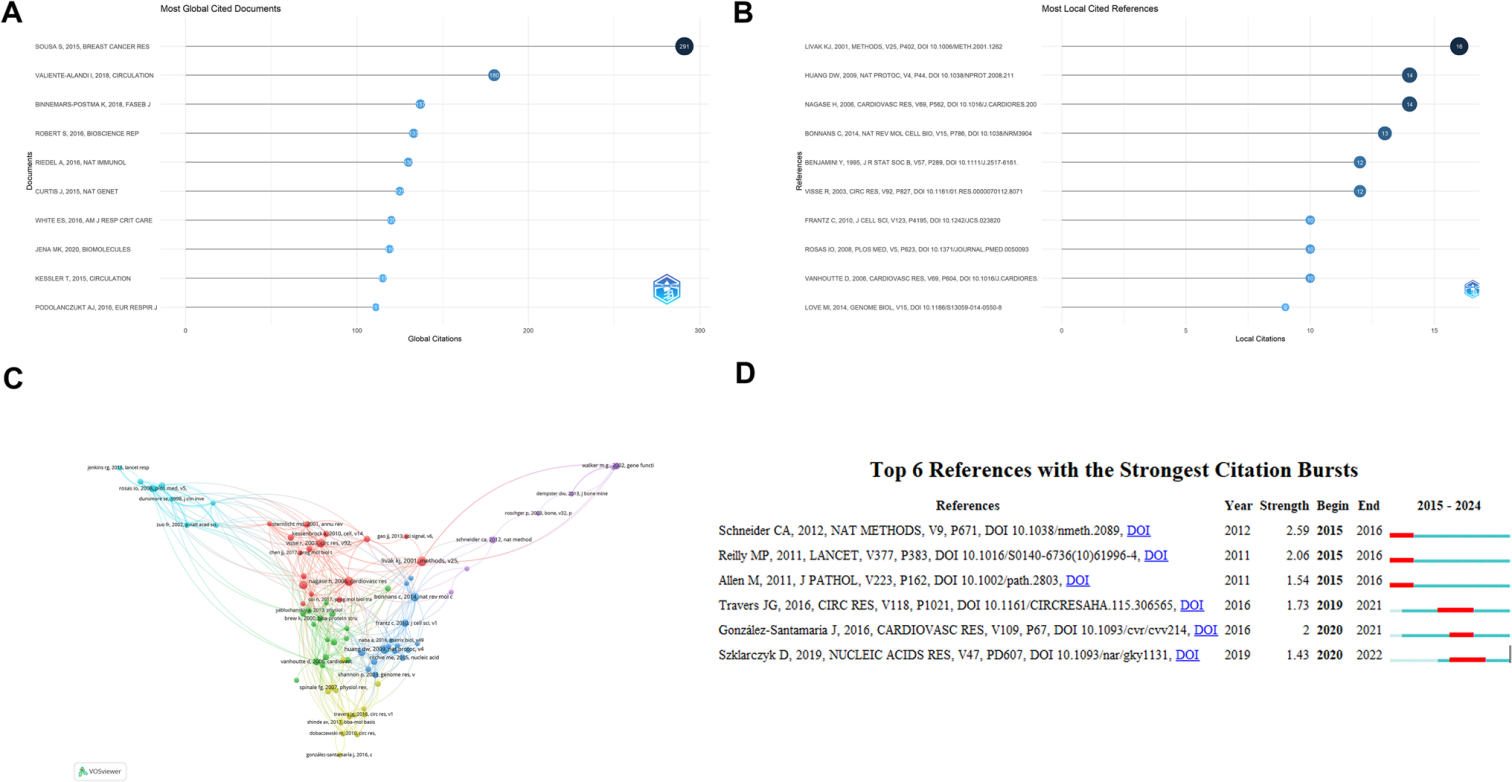
Citation and co-cited literature analysis of MXRA7 related literature between 2015 and 2024. **A** Most cited literature globally. **B** Most cited literature locally. **C** Network of co-cited literature with more than 5 citations. **D** Analysis of the “outbreak” cycle of the top 6 cited literature

The top ten most influential articles globally, identified by total citation count, provide insights into key contributions to the MXRA7 research field. Sousa S., 2015, Breast Cancer Research emerged as the most cited article, with 291 citations. Valiente-Alandi I., 2018, Circulation ranked second with 180 citations, followed by Binnemars-Postma K., 2018, FASEB Journal and Robert S., 2016, Bioscience Reports, cited 137 and 133 times, respectively (Fig. 5A).

Figure 5B highlights the most cited literature within the scope of this analysis. Livak KJ., 2001, Methods, was the most locally cited reference, cited 16 times. Its high citation count suggests its significance in research methods or tools, particularly in real-time quantitative PCR for gene expression analysis. Huang DW., 2009, Nature Protocols and Nagase H., 2006, Cardiovascular Research followed closely, each cited 14 times. The co-cited literature, analyzed using VOSviewer, revealed key nodes representing influential works. The size of each node indicates the number of co-citations, while clusters of different colors denote the strength of associations among co-cited works. Notable examples include: Livak KJ., 2001, Methods: A seminal paper on real-time quantitative PCR, widely applied in gene expression analysis, forming a central node in the graph. Nagase H., 2006, Cardiovascular Research: An impactful study on the role of MMPs in matrix remodeling, particularly in cardiovascular disease research (Fig. 5C).

CiteSpace’s burst detection algorithm was applied to identify citation bursts, defined as sharp increases in citation frequency within the dataset, and citation bursts are analysed using Kleinberg’s algorithm. The top six documents were selected based on burst intensity and duration, with a timeline indicated by a blue line and citation bursts represented by red bars (Fig. 5D). Schneider CA., 2012, Nature Methods showed the highest burst intensity (2.59). Published in Nature Methods, this article describes a widely applicable experimental or analytical method, likely related to image analysis and quantitative techniques. Reilly MP., 2011, The Lancet exhibited a burst intensity of 2.06. This high-impact paper from The Lancet is heavily cited, likely due to its focus on biomarkers or novel mechanisms in cardiovascular disease.

From the Fig. 5D, it can be deduced that literature on methodologies and tools constitutes a prominent citation hotspot. Examples include Schneider CA (2012) and Szklarczyk D (2019), which are frequently cited due to their versatility and broad applicability, making them central references within the field. Research on disease mechanisms is identified as a primary focus, as evidenced by works such as Travers JG (2016) and González-Santamaria J (2016), which delve into fibrosis and matrix remodeling in cardiovascular diseases. These studies underscore sustained research interest in this area. Additionally, the rise of data integration and analysis tools has significantly propelled research progress. The citation burst of Szklarczyk D (2019) highlights the increasing importance of bioinformatics tools and databases in facilitating high-throughput data integration, reflecting their critical role in advancing modern scientific exploration.

### Author and co‑cited author analysis

**Fig. 6:**
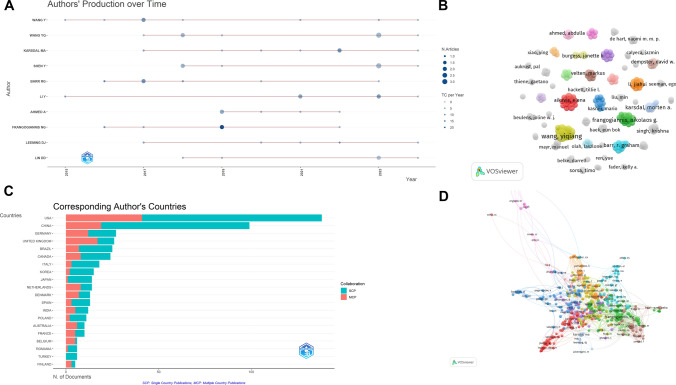
Author distribution and collaborative network analysis of MXRA7-related literature. **A** Relationship between the number of publications and the number of citations for the top 10 authors. **B** Clustering of authors with 2 or more publications. **C** Number of publications by corresponding authors in each country, categorized by single-country collaboration (SCP) and multi-country collaboration (MCP). **D** Collaborative network of authors cited more than 5 times

Figure 6A visualizes the relationship between the number of publications and citations for the top 10 authors. The length of the bars represents the annual publication output of each author, while the size of the circles indicates the total number of publications. The depth of the circle color corresponds to the number of citations in a given year. Authors such as Wang Y and Wang YQ demonstrate significant activity, with consistently high research output over the years. In contrast, between 2015 and 2017, Lin DD had a low publication volume and almost no articles were published. However, starting in 2018, Lin DD’s publication volume increased and continues to grow, suggesting that their research is gaining increasing attention. It is recommended to monitor these authors’ research directions and collaborative networks closely. In terms of time dimension, the peak research period covers the period from 2020 to 2023, especially in the areas of high-throughput technology and gene function research. This is a guide for our future research in this field. Figure 6B analyzes authors with two or more publications using VOSviewer software. Core authors, such as Wang Yiqiang and Frangogiannis NG, are established leaders in MXRA7 research, significantly contributing to the understanding of its biological functions. Their collaborative networks encompass multiple research directions. However, the relatively fewer connecting lines among authors suggest limited collaboration between these key figures, pointing to opportunities for fostering greater interdisciplinary partnerships. Figure 6C illustrates the number of papers published by corresponding authors in each country, categorized into two types of collaborations: single-country collaborations (SCP) and multi-country collaborations (MCP). The United States (USA) leads in the number of publications across both SCP and MCP categories, with a particularly pronounced dominance in SCP. China ranks second, showing a high volume of SCP papers, although its MCP count is comparatively lower. These patterns highlight the USA’s strong research leadership and China’s growing contributions, particularly through independent national efforts. China’s high SCP share reflects its independent scientific research strength in the field of MXRA7, especially in disease modelling and technical method development. However, the enhancement of multinational cooperation (MCP) will help to absorb international clinical resources and interdisciplinary experience, and accelerate the translation from basic research to clinical application. Figure 6D analyzes authors with five or more citations using VOSviewer software. Prominent authors such as Frangogiannis NG and Nagase H form a tightly interconnected collaboration network within the core areas of MXRA7 research. Conversely, some highly productive authors, such as Cowin SC and England BR, appear on the periphery of the network, suggesting that they may be focusing on independent research domains or smaller team projects despite their significant output.

This analysis underscores the importance of fostering collaborative networks among core authors and expanding multi-country collaborations to further enhance the research landscape of MXRA7.

### Keywords and research hotspot analysis

**Fig. 7:**
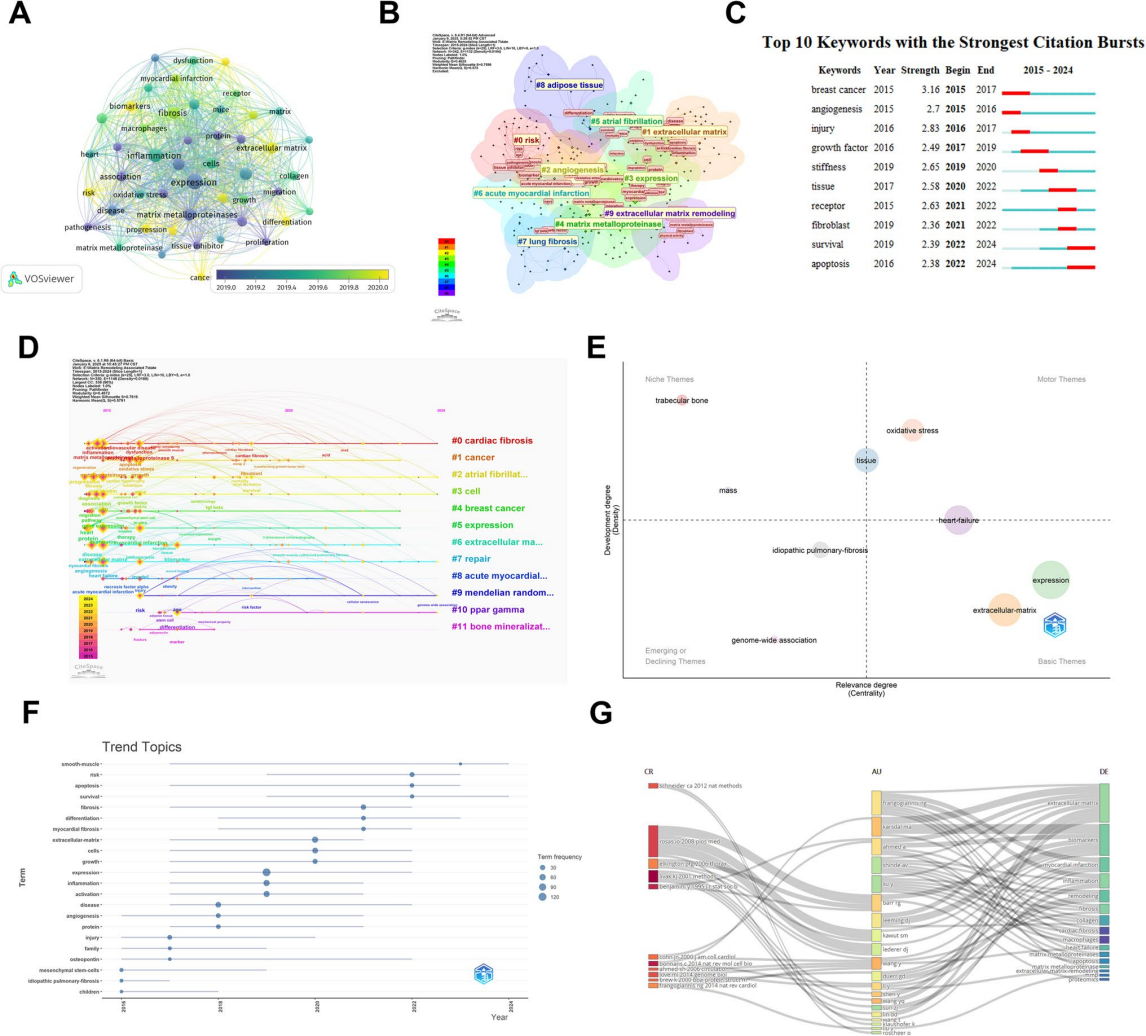
Keyword co-occurrence network and trend analysis of MXRA7 related literature. **A** Keyword co-occurrence network, showing the high-frequency keywords and their associated relationships. **B** Keyword clustering analysis, demonstrating the relationship between major research directions. **C** Keyword citation outbreaks, showing the changes of hot keywords. **D** Keyword time view, showing changes in topic activity by time. **E** Strategy map, which categorizes research topics into four quadrants and shows their trends. **F** Trending Topic Analysis, showing active keywords for recent research. **G** Sankey diagram showing the relationship between cited literature, authors and keywords

Figure 7A illustrates the high-frequency keywords in the MXRA7 research area and their co-occurrence relationships over time. Core keywords, such as “inflammation”, “extracellular matrix” and “expression” highlight the primary focus areas in this field. The time-gradient color indicates that emerging research topics include “matrix metalloproteinases” and “angiogenesis” reflecting recent hotspots in MXRA7 research. Figure 7B presents a keyword cluster analysis, identifying themes such as “risk”, “angiogenesis”, “extracellular matrix” and “fibrosis”. The interconnections between these clusters suggest overlapping research directions, such as the close relationship between “angiogenesis” and “extracellular matrix remodeling”. By categorizing these themes, the research areas related to MXRA7 can be summarized as follows:

MXRA7 and Matrix Remodeling: Themes #1 (Extracellular Matrix) and #9 (Extracellular Matrix Remodeling) emphasize the role of MXRA7 in extracellular matrix function and its involvement in related diseases. MXRA7 and Angiogenesis: Theme #2 (Angiogenesis) highlights MXRA7’s relevance to research on “oxidative stress” and “biomarkers”. MXRA7 has been identified as a key player in corneal neovascularization, particularly in inflammatory models, where it may regulate angiogenic factors to influence neovascularization processes. MXRA7 and Cardiovascular Disease: Theme #6 (Acute Myocardial Infarction) underscores the connection between MXRA7 and cardiovascular disease, reflecting the gene’s importance in this area of research. MXRA7 and Fibrosis: Theme #7 (Lung Fibrosis) focuses on the molecular mechanisms underlying fibrotic diseases and MXRA7’s role within them. MXRA7 and Inflammation/Immunity-Related Diseases: Themes #0 (Risk) and #3 (Expression) overlap significantly with inflammation-related studies. MXRA7 has shown a protective role in immune disorders, such as psoriasis. For instance, in psoriasis models, MXRA7 expression negatively correlates with key inflammatory factors like IL-17, highlighting its potential to inhibit inflammation and provide therapeutic benefits.

This analysis highlights MXRA7’s multifaceted roles in matrix remodeling, angiogenesis, cardiovascular health, fibrosis, and immune regulation, while also suggesting new avenues for future research in these intersecting areas.

Figure 7C highlights keywords with the strongest citation bursts, categorized into three periods based on research focus and intensity, Early Hotspots (2015–2017): Keywords such as “breast cancer” and “angiogenesis” were prominent during this period, with burst intensities of 3.16 and 2.7, respectively. These topics garnered significant attention, reflecting a focus on breast cancer research and neoangiogenesis. This suggests that MXRA7 genes may play a role in cancer formation and prognosis, although the precise mechanisms remain unexplored. Keywords like “injury” and “growth factor” emerged as hotspots in 2016–2017, pointing to research on trauma repair and the role of growth factors in cellular recovery. Mid-Term Hotspots (2019–2021):”Stiffness” and “tissue” became research focal points in 2019 and 2020, driven by interests in tissue engineering and biomechanics. In 2021, “receptor” and “fibroblast” gained prominence, reflecting advancements in cell signaling and fibroblast research. Near-Term Hotspots (2022–2024): The outbreak detection algorithm of CiteSpace identifies “survival” and “apoptosis” as having an emergent strength of 2.39 and 2.38, respectively (2022–2024), indicating a significant increase in their citation frequency. signaling a shift towards research on mechanisms of cell survival and apoptosis. These studies are likely linked to cancer, anti-aging, and immunotherapy applications.

Figure 7D visualizes the distribution of research topics over time using CiteSpace, with the horizontal axis representing years and the vertical axis denoting different themes. Topics such as #0 (“cardiac fibrosis”) and #1 (“cancer”) display the longest duration, indicating their sustained importance and long-term impact. Meanwhile, emerging themes like #6 (“acute myocardial infarction”) and #8 (“adipose tissue”) have garnered increasing attention in recent years. Figure 7E categorizes research themes into four quadrants: Upper Right Quadrant (Motor Themes): These are well-established, high-impact themes, exemplified by the “extracellular matrix,” which serves as the central and dominant research theme. Upper Left Quadrant (Niche Themes): These are mature but specialized themes, such as “fibrosis” and “lung fibrosis” indicating high specialization in these areas. Lower Left Quadrant (Emerging or Declining Themes): This quadrant includes topics that are either newly emerging or in decline. Lower Right Quadrant (Basic Themes): Foundational research themes, such as “angiogenesis,” are located here, underscoring their role in supporting other research directions.

An analysis of keyword trends from 2016 to 2024 revealed that long-term research areas include “smooth muscle”, “fibrosis” and “extracellular matrix” suggesting their importance in tissue regeneration, the role of the extracellular matrix, and fibrotic diseases. Meanwhile, keywords like “apoptosis” and “survival” became more active between 2022 and 2024, reflecting recent trends in cancer and cell biology. Additionally, “myocardial fibrosis” has emerged as a prominent topic in recent years, highlighting its relevance to cardiovascular disease research. This analysis underscores the dynamic evolution of research priorities in MXRA7 studies, emphasizing both long-term core themes and emerging areas of interest (Fig. 7F).

Figure 7G illustrates a three-part Sankey Diagram with three main sections: cited literature (CR), authors (AU) and keywords (DE). SanKey graph is constructed based on co-occurrence matrix, the width of the flow line indicates the strength of association between two nodes, and the connection logic is that if an author cites the left side literature in his/her paper, the two are connected by the flow line. If an author’s research topic is strongly related to the keywords on the right side, the two are connected through the streamline. The left cited literature (CR) Schneider CA 2012 Nat Methods, Livak KJ 2001 Methods literature is cited by multiple authors, associated with multiple keywords, and may be an important methodological literature in the study. Frangogiannis NG: This author is a core researcher, cited in several important papers, and also researched topics related to “extracellular matrix” and “fibrosis”. Ahmed A: another important author, closely associated with the keywords “inflammation” and “myocardial infarction”.

### Analysis of the current status of MXRA7 gene research and thoughts on future research directions

#### Exploring biological mechanisms to clinical translation

Based on existing research, the MXRA7 gene exerts significant influence in the field of cell biology. Analyzing the research trends of relevant keywords reveals that, as a critical factor in matrix remodeling, MXRA7 plays a pivotal role in the interaction between cells and the extracellular environment. It regulates fundamental biological processes such as cell migration, proliferation, and differentiation, while also participating in changes to the extracellular matrix, thus enhancing the understanding of its role in matrix remodeling [1, 2, 4, 18]. By modulating immune responses and mechanisms of wound healing, MXRA7 further deepens our understanding of cell signaling, immune responses, and tissue repair. As a negative regulator within the immune system, MXRA7 plays an essential role in the onset and progression of inflammatory diseases. As a novel potential therapeutic target, MXRA7 could provide new avenues for treating various diseases. For example, studies on its role in liver fibrosis and pulmonary fibrosis models suggest that it may influence tissue repair and inflammatory responses by regulating matrix metalloproteinase (MMP) activity, offering new therapeutic strategies for these diseases. Furthermore, MXRA7 has been shown to modulate cellular behavior during inflammation, promoting wound healing [4] and showing potential in clinical studies of cardiac fibrosis. As a key molecule in the regulation of the extracellular matrix, MXRA7 could serve as a promising therapeutic target. SUN’s research demonstrated the potential significance of MXRA7 in megakaryocyte differentiation and platelet production, offering new insights into the treatment of platelet-related diseases [20]. Additionally, Wang’s research explored the role of MXRA7 in the pathogenesis and treatment of kidney diseases, providing new perspectives on its crucial role in kidney physiology and related pathological processes, and offering novel insights into the treatment of chronic kidney disease (CKD) [31].

#### Insights on the MXRA7 study

Although the role of MXRA7 in various physio-pathological processes has been preliminarily explored, its specific mechanisms remain unclear. The interaction of MXRA7 with the extracellular matrix, its binding to different receptors, and its involvement in cell migration, proliferation, and differentiation as a matrix remodeling protein are not yet fully understood. Furthermore, the impact of different splice isoforms of MXRA7 on its function remains a key area of research. While existing literature has primarily focused on the overall expression level and tissue distribution of MXRA7, systematic studies addressing the functions of its splice variants and their roles in various diseases are lacking. In-depth investigation of MXRA7 splice variants and their functional differences in specific tissues could provide crucial insights into the diverse roles of this gene in pathological processes.

Moreover, although MXRA7 has demonstrated potential roles in several disease models, its involvement in other areas, such as tumors and cardiovascular diseases, has not been thoroughly investigated. Some studies have reported the overexpression of MXRA7 in childhood acute lymphoblastic leukemia [32, 33], but its role in other tumor types, particularly in tumor immune escape and tumor microenvironment matrix remodeling, warrants further exploration. MXRA7’s potential in inflammation regulation and extracellular matrix remodeling also suggests it could play a significant role in metabolic diseases like diabetes-related complications, particularly in delayed wound healing and tissue remodeling. Additionally, the role of MXRA7 in stem cell differentiation positions it as a promising candidate for stem cell therapies, particularly in the regulation of bone marrow mesenchymal stem cell differentiation. As research into the molecular mechanisms of MXRA7 advances, it may open new avenues for stem cell-based therapies. As a member of the matrix recombinant protein family, MXRA7 plays different roles in embryonic development, metabolic disorders, cardiovascular and neurological diseases [34–38]. Some studies have demonstrated the diagnostic significance of MXRA7 in endometriosis [39]. MXRA7 is also involved in angiogenesis and extracellular matrix repair [2], which has an important role in cancer.

Future research should focus on elucidating the molecular mechanisms underlying MXRA7’s function. High-throughput screening and molecular biology techniques will be essential for advancing our understanding of its molecular roles. Furthermore, translating these findings into clinical applications, especially in disease treatment and intervention strategies, remains a significant challenge. As our understanding of MXRA7’s function deepens, its potential as a therapeutic target for diseases such as liver fibrosis, psoriasis, and tumors should be explored. Finally, interdisciplinary approaches, particularly bridging basic and clinical research, will be crucial to uncovering the multifaceted roles of MXRA7 in diseases and its clinical applications. Collaboration between bioinformatics and clinical research will be a key trend in advancing MXRA7 research.

## Conclusion

This study presents a comprehensive bibliometric analysis of the literature on the MXRA7 gene published between 2015 and 2024, highlighting the research trends and emerging hotspots in this field. From a publication volume perspective, MXRA7 research has shown a consistent upward trajectory, particularly since 2017, with a significant increase in the number of studies. This surge reflects growing recognition of the gene’s research value in various biological processes. The United States leads in MXRA7 research, both in terms of publication volume and citation count, underscoring its central role in the global academic community. China and the UK also contribute significantly to this field, with China showing notable growth in recent years, emerging as the second-largest contributor after the US.

From a journal perspective, MXRA7 research is predominantly published in high-output journals such as PLOS ONE, which further accelerates the dissemination and development of research in this area. Co-citation analysis reveals that foundational journals in basic biology and cellular research, including Journal of Biological Chemistry and Nature, have exerted a major influence on MXRA7 research, reflecting the gene’s close connection with these fields. Furthermore, keyword analysis identifies terms such as “inflammation,” “extracellular matrix,” “matrix metalloproteinases,” and “angiogenesis” as central to the research landscape, indicating MXRA7’s key roles in immune response, tissue repair, and fibrosis.

Research by influential authors like Frangogiannis NG and Ahmed A has significantly advanced understanding of MXRA7’s involvement in cardiovascular diseases and matrix remodeling, providing foundational theoretical support for its broader implications.

Despite these advances, research on MXRA7 remains an evolving field and faces several challenges. Future studies should prioritize in-depth investigations into the molecular mechanisms of MXRA7, particularly its roles in various disease models. Additional focus should be given to understanding the function of MXRA7’s splice variants, their involvement in immune responses, tumors, and other diseases, as well as promoting the clinical application of MXRA7 in precision medicine. With the continued resolution of these issues, MXRA7 is poised to emerge as a therapeutic target for a wide range of diseases. potentially offering novel avenues for clinical treatment.

## Strengths and limitations

This study systematically analyzed the literature related to the MXRA7 gene using bibliometric methods. However, several limitations should be acknowledged. First, Data retrieval for this study was based solely on the Web of Science (WoS) database. Despite the authority of WoS in the field of high-quality literature inclusion and citation analysis, the use of a single database may result in the omission of some relevant literature. Future research could further improve the comprehensiveness of literature coverage and reduce potential selection bias by integrating multi-source databases such as Scopus and Dimensions. Although we strictly adopted “Matrix Remodeling Associated 7” and “MXRA7” as search terms based on the MeSH thesaurus, we may have missed studies that used non-standard aliases, ignored the synergistic or comparative analyses between MXRA7 and other genes, and therefore future studies still need to further refine the search terms. or comparative analyses of MXRA7 with other genes, and therefore future studies need to further refine the search terms. Additionally, the bibliometric analysis relied on keywords and citation data, which, although effective in identifying research trends and hotspots, may overlook in-depth discussions or smaller-scale scientific breakthroughs. Moreover, this method does not fully account for the quality or impact of the literature, as the number of citations and publications alone may not accurately reflect the innovation or significance of the research. Finally, due to delays in the database’s data update cycle, some of the most recent literature may not have been included in the analysis, which could affect the timeliness and completeness of the findings.

## Data Availability

All the data can be obtained from the open-source website we provide, and the conclusion can be drawn through the analysis of the relevant software.
