# Correction: Bibliometric analysis of MXRA7 gene research trajectory: trends and insights (2015–2024)

**DOI:** 10.1007/s12672-025-03016-x

**Published:** 2025-07-17

**Authors:** Huihui Zhang, Ying Shen, Peijian Bai, Xiaorui Wu, Ping Li, Ting Wang

**Affiliations:** 1 Oncology Department of Integrated Traditional Chinese and Western Medicine, The First Affiliated Hospital of Anhui Medical University, Hefei, 230001 China; 2 National Clinical Research Center for Hematologic Disease, Institute of Blood and Marrow Transplantation of Soochow University, The First Affiliated Hospital of Soochow University, Suzhou, 215006 China

**Correction: Discover Oncology (2025) 16:989** 10.1007/s12672-025-02824-5

The original publication of this article contained a typo in the abstract. The incorrect and correct information is listed in this correction article, the original article has been updated.

Incorrect

Key studies appear in high-impact journals like PLOS ONE, and influential authors such as Frangogiannis NG have propelled the field.

Correct

Key studies appear in high-impact journals like PLOS ONE, and influential authors such as Wang Yiqiang have propelled the field.

